# Using Automatic Speech Recognition to Assess Thai Speech Language Fluency in the Montreal Cognitive Assessment (MoCA)

**DOI:** 10.3390/s22041583

**Published:** 2022-02-17

**Authors:** Pimarn Kantithammakorn, Proadpran Punyabukkana, Ploy N. Pratanwanich, Solaphat Hemrungrojn, Chaipat Chunharas, Dittaya Wanvarie

**Affiliations:** 1 Department of Computer Engineering, Faculty of Engineering, Chulalongkorn University, Bangkok 10330, Thailand; 6270194021@student.chula.ac.th; 2 Department of Mathematics and Computer Science, Faculty of Science, Chulalongkorn University, Bangkok 10330, Thailand; naruemon.p@chula.ac.th (P.N.P.); dittaya.w@chula.ac.th (D.W.); 3 Chula Intelligent and Complex Systems Research Unit, Chulalongkorn University, Bangkok 10330, Thailand; 4 Department of Psychiatry, Faculty of Medicine, Chulalongkorn University, Bangkok 10330, Thailand; solaphat@hotmail.com; 5 Cognitive Fitness and Biopsychological Technology Research Unit, Chulalongkorn University, Bangkok 10330, Thailand; 6 Cognitive Clinical & Computational Neuroscience Research Unit, Department of Internal Medicine, Faculty of Medicine, Chulalongkorn University, Bangkok 10330, Thailand; chaipat.c@chula.ac.th; 7 Chula Neuroscience Center, King Chulalongkorn Memorial Hospital, Thai Red Cross Society, Bangkok 10330, Thailand

**Keywords:** MoCA, ASR, speech recognition, scoring, language fluency test, Thai tonal language, LOTUS corpus

## Abstract

The Montreal cognitive assessment (MoCA), a widely accepted screening tool for identifying patients with mild cognitive impairment (MCI), includes a language fluency test of verbal functioning; its scores are based on the number of unique correct words produced by the test taker. However, it is possible that unique words may be counted differently for various languages. This study focuses on Thai as a language that differs from English in terms of word combinations. We applied various automatic speech recognition (ASR) techniques to develop an assisted scoring system for the MoCA language fluency test with Thai language support. This was a challenge because Thai is a low-resource language for which domain-specific data are not publicly available, especially speech data from patients with MCIs. Furthermore, the great variety of pronunciation, intonation, tone, and accent of the patients, all of which might differ from healthy controls, bring more complexity to the model. We propose a hybrid time delay neural network hidden Markov model (TDNN-HMM) architecture for acoustic model training to create our ASR system that is robust to environmental noise and to the variation of voice quality impacted by MCI. The LOTUS Thai speech corpus was incorporated into the training set to improve the model’s generalization. A preprocessing algorithm was implemented to reduce the background noise and improve the overall data quality before feeding data into the TDNN-HMM system for automatic word detection and language fluency score calculation. The results show that the TDNN-HMM model in combination with data augmentation using lattice-free maximum mutual information (LF-MMI) objective function provides a word error rate (WER) of 30.77%. To our knowledge, this is the first study to develop an ASR with Thai language support to automate the scoring system of MoCA’s language fluency assessment.

## 1. Introduction

Cognitive decline is a common health issue among aging populations [1]. Memory loss and forgetfulness are part of the normal aging process; however, memory loss that affects daily life can be a symptom of dementia [2]. Neurodegeneration is a common cause of dementia [3]; its symptoms vary among individuals and include memory loss, deterioration of speech, motor skills, and cognitive function [4]. Mild cognitive impairment (MCI) is a condition between normal age-associated memory impairment and dementia [5]. It causes cognitive problems that are noticeable to individuals and those close to them, but does not impact daily life activities. MCI is believed to be a high-risk condition for the development of Alzheimer’s disease (AD) [6], and the early detection of MCI allows for health professionals to better plan for and treat individuals at risk of developing AD or other types of dementia.

Numerous screening tools have been developed for detecting dementia. Among them, the mini-mental state examination (MMSE) [7] is the most widely used, but difficulties have been reported in detecting early dementia. To address this problem, the Montreal cognitive assessment (MoCA) was developed to screen patients with MCI, while performing on a normal range of MMSE [8]. The MoCA showed a sensitivity of 90% whereas the MMSE had a sensitivity of 18% in identifying MCI patients with memory loss [9]. MoCA assesses several parts of brain function, such as short-term memory, visuospatial abilities, executive function, attention, concentration and working memory, language, and orientation; it is available in both paper and digital formats. However, data collection and scoring require extensive assistance by health professionals, and further data analysis is limited to text input only.

MoCA is available in two versions: a standard paper-and-pencil version and an electronic version. Using the electronic version enables opportunities for utilizing technology such as artificial intelligence (AI) to significantly improve the efficiency and quality of cognitive assessment. To address the limitations of the standard MoCA test procedure for Thai, researchers from the Faculty of Medicine in collaboration with the Department of Computer Engineering at Chulalongkorn University initiated a new project to develop a customized version of MoCA as an iPad application called “Digital MoCA”, which provides Thai language support. Their main goal was to enable automatic data collection and utilize machine learning techniques for the early detection of MCI patients in Thailand.

Digital MoCA is based on the standard MoCA test criteria with Thai language support, including automatic data collection and scoring with limited assistance from health professionals. The automatic data collection of voice recording and drawing input enable further data analysis using machine learning techniques for better understanding MCI risk factors. Since the composition of this paper, digital MoCA is still in the early phase of development with a prototype version under evaluation by a group of physicians and a health professional team. This study is a sub-project of the project initiative to utilize new AI techniques for MCI detection improvement.

Digital MoCA’s automatic data collection of voice recording and drawing input enable further data analysis using machine learning techniques to better understand MCI risk factors.

To achieve reliable results through digital MoCA, automated scoring is preferred for limiting the impacts of human error, personal judgment, and bias. Automatic speech recognition (ASR) can be adopted to translate voice recordings into word sequences for automated scoring and evaluation. This study focuses on applying ASR techniques to detect proper Thai words to assist the automated scoring system of the language fluency test of the MoCA and reduce the need for health professionals providing personal judgments during assessments; this will help to increase the overall reliability of MoCA scores. Developing ASR for intended functionalities in this situation comes with several challenges, such as the great variety of pronunciation, intonation, tone, and accent of the patients—all of which might differ from healthy controls. For example, many patients with MCI have slurred speech or a muffled voice, which are somewhat unintelligible to the human ear and have a direct impact on the quality of recognition. Thai is a tonal language in which the pronunciation of isolated words outside a specific context markedly increases the difficulty of detecting the correct tone, especially in older adults with MCI and unclear speech. Furthermore, Thai accents differ by region, and this may affect the accuracy of word detection when developing ASR with a small dataset. Additionally, background noises and conversations between patients and health professionals are recorded during the test. To overcome these challenges, several techniques need to be applied in combination with a special algorithm to differentiate between words that are eligible for MoCA scoring versus those that are not.

In our study, the ASR system plays an important role in enabling reliable word detection for the MoCA language fluency test scoring system. Since Thai is a tonal language, the accuracy of the ASR system depends highly on reliability of tone detection. Understanding tone in the Thai language system can help determine a proper tone mark in the lexical model of ASR system development.

The Thai language system is composed of consonant, vowel, and tonal sounds. These are described in the form of/C_i_VC_f_^T^/or/C_i_V^T^/, where C_i_ is the initial consonant, V is a vowel, C_f_ is the final consonant, and T represents the tone level. C_i_ can either be a single or a clustered consonant, whereas V can either be a single vowel or a diphthong [10].

**Figure 1:**
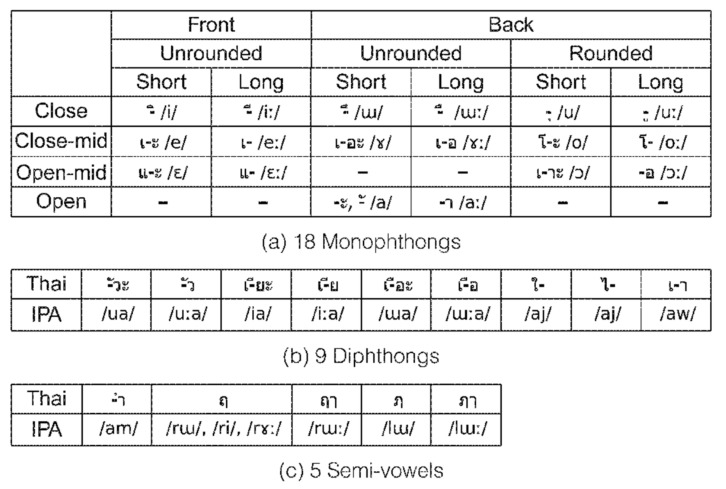
Thirty-two vowels in Thai: (**a**) 18 monophthongs, (**b**) 9 diphthongs, and (**c**) 5 semi-vowels.

There are 44 consonant symbols in Thai that represent 21 initial consonant and 8 final consonant sounds (Table 1), wherein 18 vowel symbols in combination with 3 consonants are used to create 32 vowels (Figure 1) [11].

**Figure 2:**
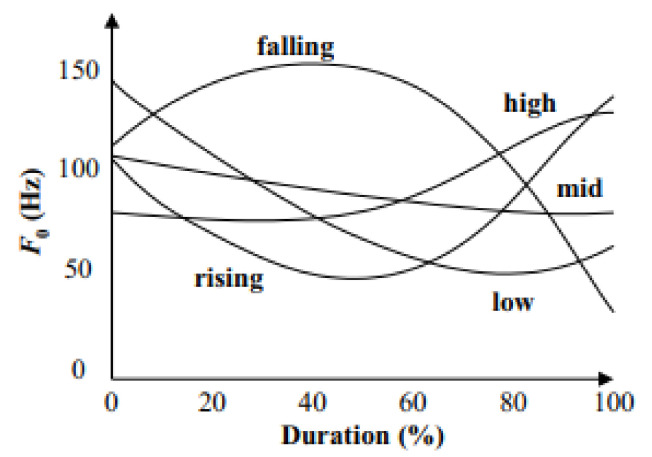
*F*_0_ contours of the five Thai tones.

There are five lexical tones in Thai as follows: mid/M/, low/L/, high/H/, falling/F/, and rising/R/. The identification of tone relies on the shape of the fundamental frequency (F0) contour (Figure 2) [10]. A change in the tone will change the meaning of a word. For example, khaa has five meanings according to the effect of the tone (Table 2).

Our study aims to develop an ASR model for MCI patients that can work well with a limited amount of training data. The key contributions of this research are the techniques that we used to develop an ASR model that can support the Thai speech data of MCI patients from a very small sample dataset with an acceptable word error rate (WER). Achieving similar accuracy would normally require a very large amount of MCI patient speech data for training using the standard ASR technique. We discovered that using a phonetically distributed dataset from the LOTUS corpus with MCI speech data helped the ASR to learn the speech features of all the basic phone units in Thai as part of the TDNN-HMM model training. Consequently, the ASR model could successfully recognize Thai words outside the training dataset and reduce the need for manual data collection to build a large MCI patient training dataset. The main novelty of this study is the decoding, where a new algorithm was developed for the detection of eligible Thai words to automate the fluency score calculation. This may be extended and used to support other languages in the near future.

The remainder of this paper is organized as follows: Section 2 reviews the related works on ASR systems for speech analysis, verbal fluency assessment, and tonal language support. Section 3 describes the data pre-processing and augmentation procedure with the experiment setup using several GMM-HMM model training approaches as the baseline. The TDNN model architecture for end-to-end LF-MMI is proposed with evaluation metrics. Section 4 and Section 5 present the experimental results and discuss them, respectively. Finally, the Conclusions are presented in Section 6.

## 2. Related Work

Many studies have applied speech processing to cognitive assessment. The common approach used for analysis relies on acoustic features from speech data and text features extracted through ASR. König et al. [13] analyzed dementia-related characteristics from voice and speech patterns by developing a classifier using a support vector machine (SVM), with features extracted from spoken tasks. These tasks were characterized by the continuity of speech using the duration of contiguous voice and silent segments as well as the length of contiguous periodic and aperiodic segments to derive statistical values as vocal features. For the semantic fluency task, the vocal feature was defined as the distance in time from the first detected word to each following word. The evaluation results show the following classification accuracy: healthy control (HC) versus MCI, 79%; HC versus AD, 87%; and MCI versus AD, 80%. These findings suggest that automatic speech analyses could be an additional assessment tool for older patients with cognitive decline.

Spontaneous speech can provide valuable information about the cognitive state of individuals [14]; however, to retrieve useful clinical information, it needs to be transcribed manually. Zhou et al. [14] proposed an ASR system for generating transcripts automatically and extracting text features to identify AD with an SVM classifier. They used an open-source Kaldi ASR toolkit [15] to optimize performance for the speakers with and without dementia based on the dementia bank (DB) corpus through insertion penalty and language model weight adjustment. They obtained an average WER of 38.24%, showing an improvement over their previous work, which had employed commercial ASR. However, their results were limited by the poor quality of audio in the DB corpus, thus necessitating further exploration.

Language fluency tests are a main task in cognitive tests; in the literature, they are also known as verbal fluency (VF) tasks, which refer to short tests of verbal functioning where patients are given 1 min to produce as many unique words as possible within a sematic category (semantic fluency) or start with a given letter (phonemic fluency) [16]. In clinical practice, VF tests are administered manually; few studies have evaluated computerized VF administration and scoring. Pakhomov et al. [17] applied ASR to speech data collected during VF tasks to obtain an approximate count of legitimate words. They implemented an ASR system based on Kaldi’s [15] work using a speaker-independent acoustic model with a specially trained animal fluency language model and applied confidence scoring to the post-process ASR output. Standard manual scoring was performed, including transcribing all responses during the VF task to be used for evaluating the ASR decoder performance. They achieved a WER of 56%, which was relatively high; however, the results suggested that the combination of speaker adaptation and confidence scoring improved overall accuracy and was able to produce a VF estimated score that was very close to the ones yielded by human assessment.

Tröger et al. [18] proposed telephone-based dementia screening with automated semantic verbal fluency (SVF) assessment. Speech was recorded through a mobile tablet built-in microphone and downsampled to 8 KHz to simulate telephone conditions. SVF sound segments were analyzed using Google’s ASR service for possible transcriptions. Various features were extracted from generated transcripts and evaluated using an SVM classifier. The overall error rate of the automatic transcripts was 33.4%, and the automated ASR classifier reached results comparable with those of the classifier that utilized manual transcriptions. Lauraitis et al. [19] proposed neural impairment screening and self-assessment using a mobile application for MCI detection based on the self-administered gerocognitive examination (SAGE) screening. They developed a mobile application to collect data from different tasks. A VF task was conducted by instructing participants to write down 12 different items in a given category as text field in the mobile application for calculating the SAGE score. Voice recording was performed as an additional task to evaluate speech impairment by extracting several features, including pitch, mel-frequency cepstral coefficients (MFCC), gammatone cepstral coefficients, and spectral skewness, for further speech analysis with an SVM and a bidirectional long short-term memory classifier that had 100% and 94.29% accuracy, respectively.

Various approaches have been proposed for ASR with Thai language support over the years. For instance, Chaiwongsai et al. [12] proposed HMM-based isolated word speech recognition with a tone detection function to improve the accuracy of ASR for tonal languages. The tone detection function was added as a parallel computation process to detect tone level and map the results from speech recognition to obtain the final results. Experimental results revealed that the accuracy of Thai word detection improved by 4.94% for TV remote control commands and by 10.75% for Thai words that had different meanings with each tone.

One approach to avoid new training steps whenever new words are added into the dictionary is the phoneme recognition approach. To this end, Theera-Umpon et al. [20] proposed a new method to classify the tonal accents of syllables using soft phoneme segmentation techniques for Thai speech, which was better than the hard-threshold approach for phoneme classification. Hu et al. [21] conducted an experiment with Mandarin and Thai words by incorporating tonal information from fundamental frequency (F0) and fundamental frequency variation into the convolution neural network (CNN) architecture and compared CNN with DNN. The WER for Thai was 33.19% and 35.16% for CNN and DNN.

## 3. Materials and Methods

**Figure 3:**
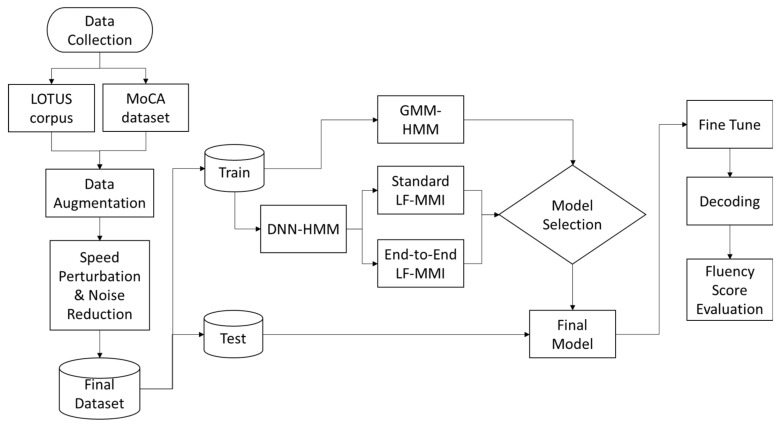
Overall experiment methodology.

In this section, we proposed the end-to-end ASR model with a special decoding algorithm to support the language fluency score. The overall experiment methodology and data processing flow are depicted in Figure 3.

### 3.1. Data Collection

**Figure 4:**
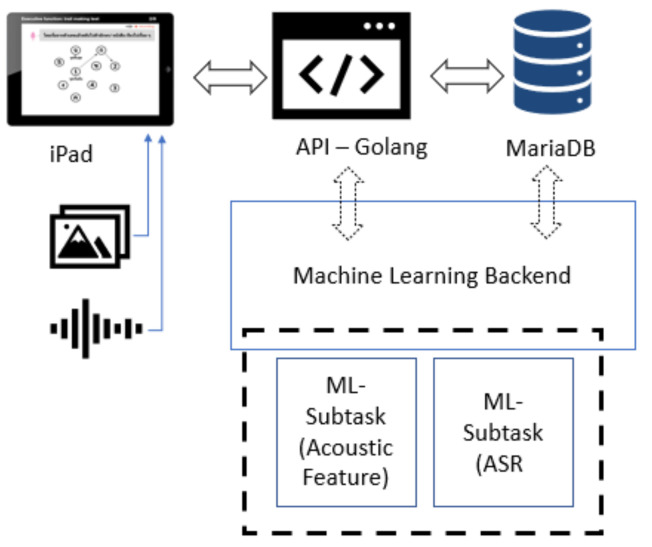
Digital MoCA system architecture overview.

Speech data were collected as part of the digital MoCA project trial run in Chulalongkorn Hospital, Thailand, with 90 participants (52 women and 38 men) aged 60–80 years. During the language fluency test, each patient was given 1 min to orally produce as many Thai words as possible beginning with “ก“ (/k/). The whole utterance was recorded as a single audio file through a standard built-in microphone on an iPad device with a sampling rate of 12 kHz in an M4A file format and stored in the digital MoCA database (Figure 4).

### 3.2. Data Preprocessing

Several challenges became apparent in the speech data; for example, variations in the pronunciation of each patient, mixtures of conversation sentences with the intended word and background noise, and the sound of the physician’s digital pencil touching the iPad screen impacted the training dataset and accuracy of speech recognition. Several audio files that contained conversations between the patient and physician during the test were manually removed before the data were processed further.

**Figure 5:**
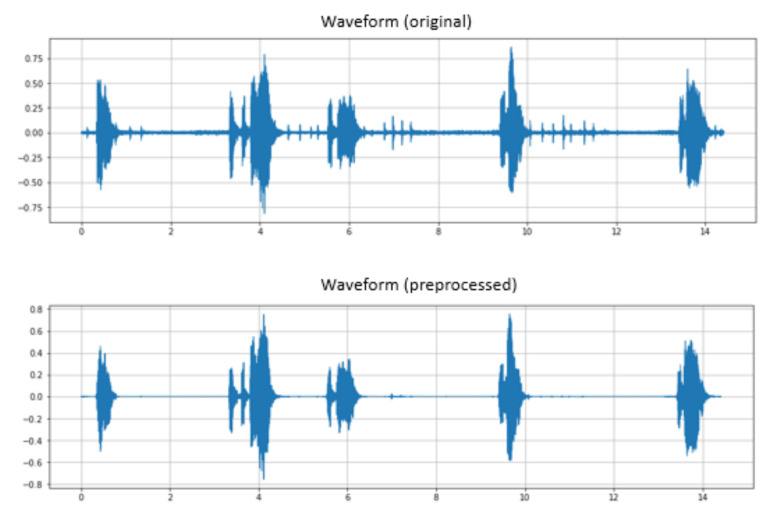
Patient’s speech waveform after preprocessing.

To improve the data quality, we implemented preprocessing steps for the original speech data by applying a recurrent neural network noise suppression algorithm (RNNoise) [22] to remove the background noise from all utterances with an additional spectral gating filter to eliminate digital pencil sounds and generate clean speech data (Figure 5).

### 3.3. Speech Corpus and Data Augmentation

We did not have a sufficient amount of speech data for Thai words beginning with “ก” to develop a reliable ASR system for this task. We incorporated the large vocabulary Thai continuous speech recognition corpus called LOTUS [23] into the training data. The LOTUS corpus consists of two principle datasets: the phonetically distributed (PD) set and the 5000 item vocabulary set. The PD set contains most of the phonemic units that occur in the language, while the 5000-item vocabulary set contains 3 subsets: the training set (TR), development test set (DT), and evaluation test set (ET). In our study, the PD set was used for acoustic model training to cover most of the basic phone units in Thai. As a result of the limited amount of data, we performed the augmentation process in two stages. The first stage involved using a pitch shift with a high step frequency to simulate data garnered from different participants. Therefore, we needed to ensure that our dataset contained a greater variety of pitch, while in the latter stage an online augmentation was performed to increase the speed and pitch of utterance, with increased iterations using a lower step as part of the model training. With these two stages of augmentation, we expected to make our model more generalizable under the constraints of limited amounts of training data.

Data augmentation using frequency shifts at 100 Hz, 300 Hz, and 500 Hz was applied to both the original and clean speech data. Additional speed perturbation to generate more data with higher and lower speeds not more than 12% of the original speed was implemented to populate the high-resolution training dataset. The details of the training dataset are summarized in Table 3.

### 3.4. Model Training

The hidden Markov model (HMM) was the main foundation for speech recognition for decades [24]; further, the Gaussian mixture model hidden Markov model (GMM-HMM) system was a robust model used to develop the ASR system for many years. A novel hybrid model architecture called the deep neural network hidden Markov model (DNN-HMM) was demonstrated to outperform GMM-HMM on various speech recognition benchmarks [25] and was widely used in speech recognition recently. However, achieving a good accuracy of DNN training normally requires a large amount of data, which is not available in our domain. To explore an optimal solution with the existing constraints, we developed an ASR system based on Kaldi [15] and created two training models using the standard GMM-HMM and DNN-HMM for comparison.

### 3.5. GMM-HMM Acoustic Model

**Figure 6:**
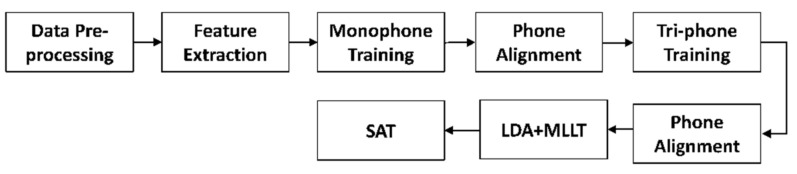
GMM-HMM model training sequence.

Since Thai is a tonal language, we extracted MFCC together with pitch features to help improve the system’s overall performance [26]. After these features were extracted, we then transformed MFCC and pitch features with cepstral mean and variance normalization following the training sequence shown in Figure 6.

In addition to the standard acoustic model mono-phone and tri-phone training, linear discriminant analysis (LDA), maximum likelihood linear transform was applied to reduce feature vectors and derive the unique transformation for each speaker, followed by normalization with speaker adaptive training to reduce inter-speaker variability in the training data [27].

### 3.6. DNN-HMM Acoustic Model

**Figure 7:**
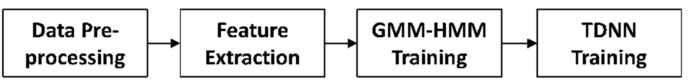
LF-MMI model training sequence.

Several types of DNN architectures are supported by Kaldi, among which the most popular is TDNN [28]. It uses a lattice-free version of the MMI objective function called chain models, which use smaller frame rates at the output of the neural net to reduce computation at the test time, making the results faster to decode. The standard chain models training sequence relies on alignment data from the GMM-HMM stage as input to the TDNN layers (Figure 7).

**Figure 8:**
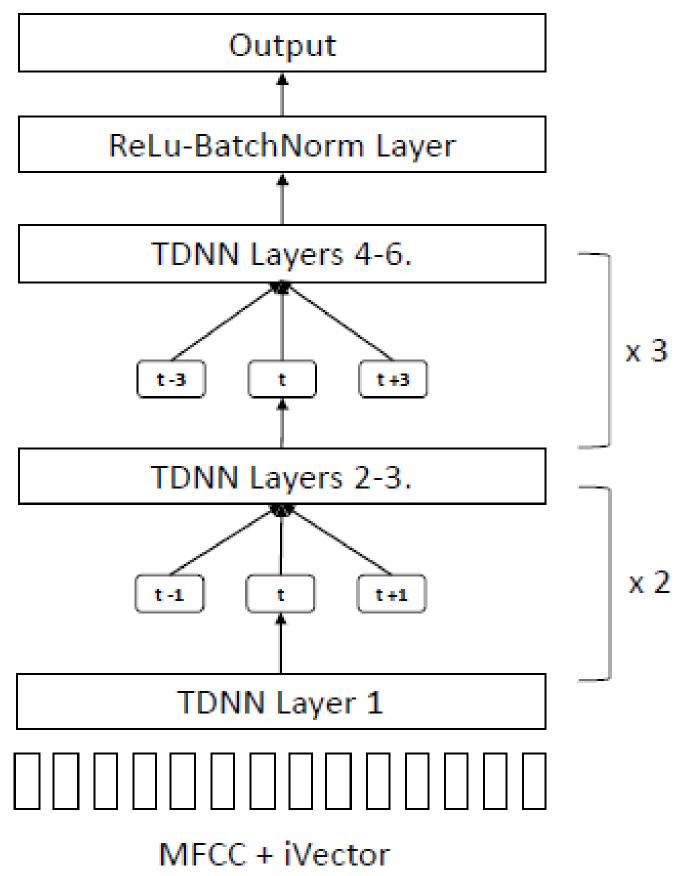
TDNN architecture for LF-MMI.

The architecture of the LF-MMI model comprises five TDNN layers with splicing indexes as (−1,0,1) (−1,0,1) (−3,0,3) (−3,0,3) (−3,0,3), as shown in Figure 8; (−1,0,1) means that the TDNN layer will process the input vectors at time t − 1, t, and t + 1, and (−3,0,3) means the TDNN layer will process input vectors at time t − 3, t, and t + 3.

**Figure 9:**
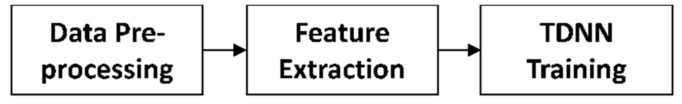
EE-LF-MMI model training sequence.

As a result of the limited amount of digital MoCA training data, the quality of the GMM-HMM stage may not produce acceptable results with our chain models. We used another technique proposed by Hadian et al. [29] called end-to-end LF-MMI (EE-LF-MMI), where training was performed in a flat-start manner using a single DNN in one stage without using any previously trained model or alignment data (Figure 9).

**Figure 10:**
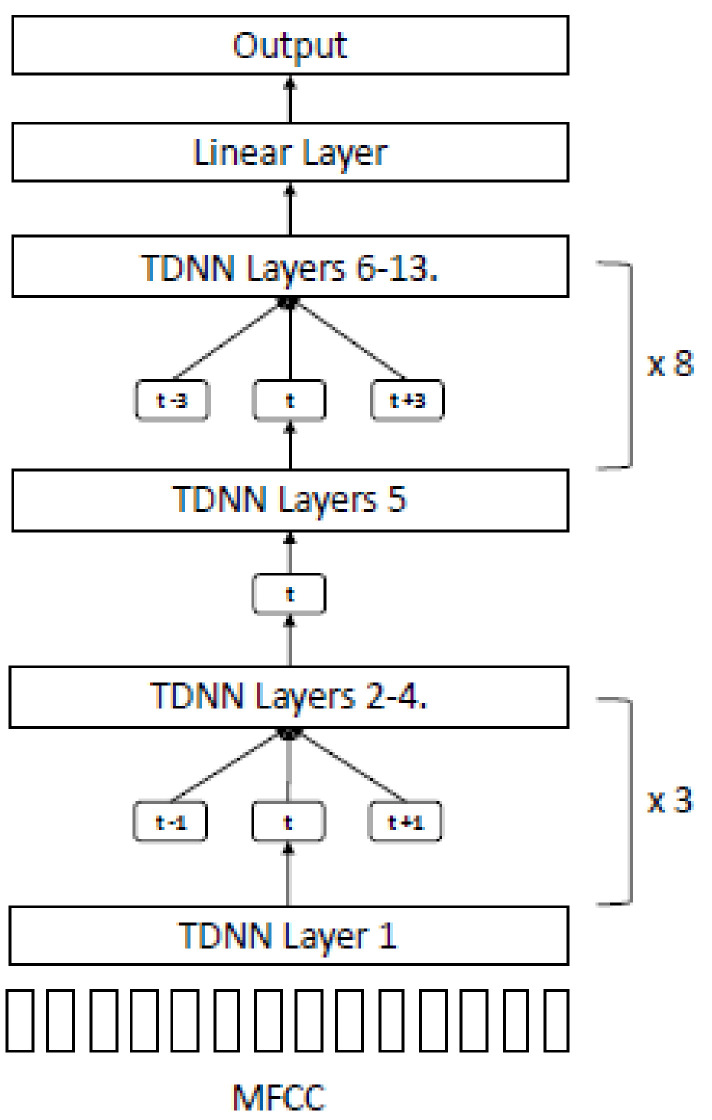
TDNN architecture for EE-LF-MMI.

The model architecture for end-to-end LF-MMI comprised 13 TDNN layers with splicing indexes (−1,0,1) in layers 2–4 and (−3,0,3) in layers 6–13 (Figure 10).

### 3.7. Language Model and Lexicon

A special dictionary was prepared by extracting all words starting with “ก” from a standard Thai dictionary, yielding 2253 words. We combined all the utterance transcriptions from the LOTUS corpus and the digital MoCA language fluency test dataset, as well as the special dictionary to construct a text corpus for tri-gram language model training and lexicon creation.

### 3.8. Evaluation Metrics

The performance of ASR systems is usually evaluated by WER, which is calculated as follows in (1):

(1) $$\mathrm{WER}=\frac{\left(I+S+D\right)}{N}$$

where *I* is the number of insertions, *S* is the number of substitutions, *D* is the number of deletions, and N is the number of words in the reference.

To evaluate the performance of the MoCA language fluency scoring, we defined a new metric called fluency score accuracy (FSA), to measure the difference between the final score rated manually by health professionals and the score calculated by the system. This is shown in (2) below:

(2) $$\mathrm{FSA}=\frac{TM}{\left(TM+TN\right)}$$

where *TM* is the total number of utterances that received the same score in the manual and automatic calculations, and *TN* is the total number of utterances that received different final scores in the manual and automatic calculations.

### 3.9. Decoding and Scoring

According to the scoring criteria of the MoCA test for language fluency, scores are given to words that start with “ก” as defined in a standard Thai dictionary, excluding proper names and duplicate words. The final score is 1 if the total eligible word count is 11 or higher; otherwise, the final score is 0. Thus, the final score function can be calculated as shown in (3):

(3) $$\mathrm{Fluency}\;\mathrm{score}\;f\left(x\right)=\left\{\begin{array}{c} 0,\;x<11\\ 1,\;x\geq 11 \end{array}\right.$$

where x is the total number of unique words in the test utterances.

**Figure 11:**
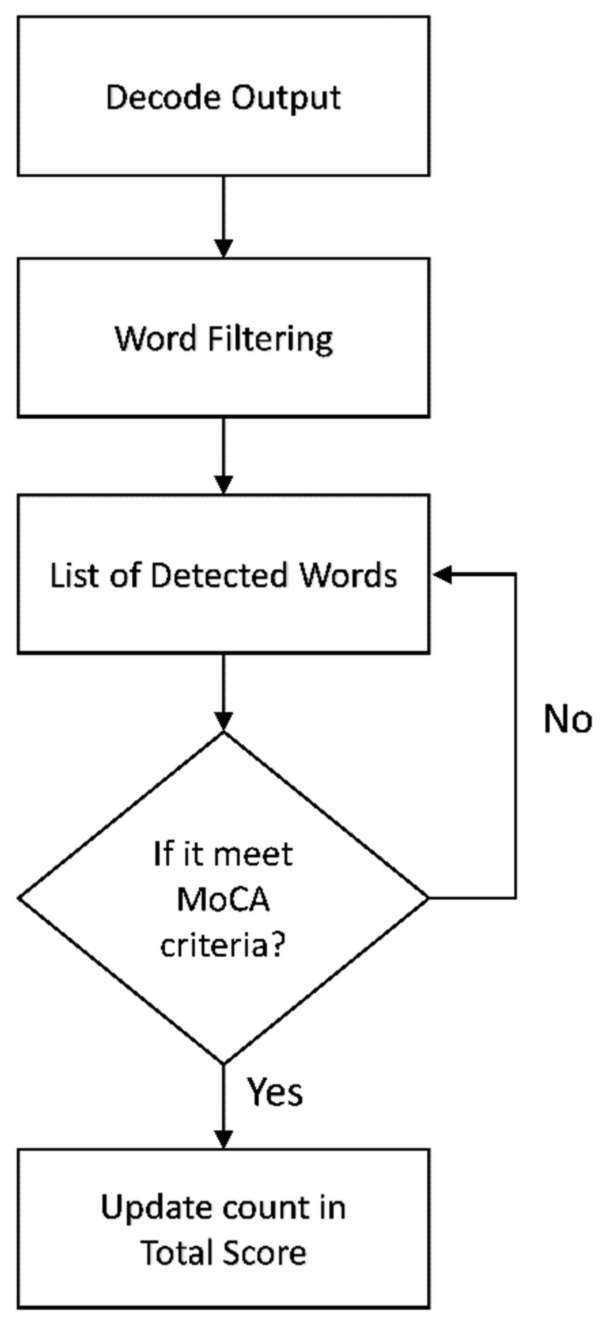
Decoding and scoring flowchart.

As output word sequences might contain ineligible words because of recognition process errors as well as unintended spoken words from patients, we implemented additional steps to filter words that begin with the initial phoneme/k/corresponding to “ก” in Thai before feeding them into the scoring algorithm. The steps for decoding and scoring are illustrated in Figure 11.

## 4. Results

### 4.1. Automatic Speech Recognition Results

We conducted an experiment with the original data from the digital MoCA and LOTUS corpus using the GMM-HMM model as the baseline to confirm our hypothesis: incorporating data from the LOTUS corpus can improve the accuracy of our model. We performed GMM-HMM training with the same configuration applied to each dataset from digital MoCA, LOTUS, and a combined set from both corpora. We divided a combined set into five folds to create a training and validation set for evaluation, using cross-validation on each fold owing to the limited sample of digital MoCA within the dataset. The results show that our GMM-HMM model achieves good accuracy for the LOTUS training samples, but does not work well with the data collected from digital MoCA because of the smaller sample size, while the combined dataset from LOTUS and digital MoCA improves the overall accuracy of the model when validated with data from digital MoCA (Table 4).

To evaluate the performance of our proposed model, we performed an experiment with the combined dataset with data augmentation using different configurations and features for GMM-HMM, TDNN-HMM (LF-MMI), and TDNN-HMM (EE-LF-MMI). The results show that the proposed model EE-LF-MMI improves the overall accuracy of the recognizer without a need for alignment data from the previous training in the GMM-HMM model (Table 5).

In our proposed model, we followed the architecture of EE-LF-MMI, as shown in Figure 8. However, we made some modifications using a different number of TDNN layers and hyperparameter tuning to discover the best setting for our environment (Table 6). The final configuration of our proposed model (EE-LF-MMI-4) achieved WER at 30.77% using 13 TDNN layers with an acoustic weight at 0.8. Furthermore, our final settings could reduce the training time by 50% with improved accuracy from the baseline.

### 4.2. Data Augmentation Analysis

As the amount of our data was limited, we performed augmentation in two stages. The first stage was using pitch shift with a high step frequency to simulate data from different participants, so we needed to ensure that our dataset contained more variety of pitch. In the later stage, an online augmentation was performed to increase the speed and pitch of an utterance with more iterations using a lower step as part of the model training. With these two augmentation stages, we expect to make our model more generalizable with limited training data.

We conducted a comparison to understand the impact of the data augmentation techniques used in our proposed model. We applied a combination of frequency shift and noise reduction and incorporated the LOTUS corpora to help reduce errors and improve overall accuracy (Table 7).

### 4.3. Word Count and Recoginition Result Analysis

We analyzed the results of the decoding output from our model for each speaker to determine the source of the errors. Substitution error was the main contributor, with an average of 24.18%; 0.82% of the problem originated from insertion and 5.77% from deletion errors, while the percentage of words that could be detected correctly was in the range of 70.05% (Table 8).

**Figure 12:**
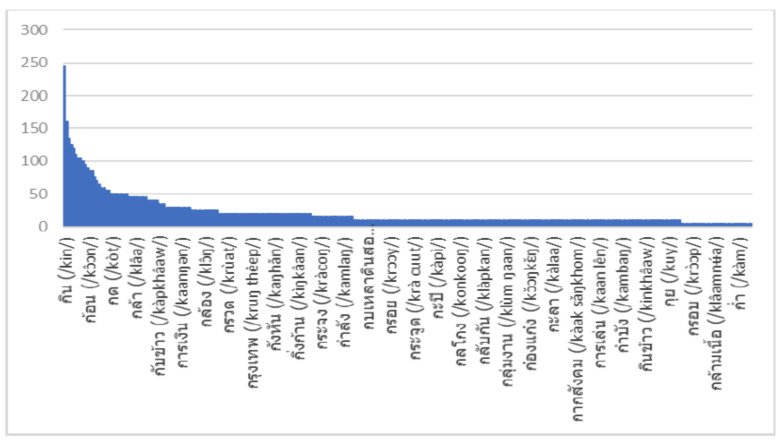
Word count from the MoCA training data.

We conducted further analysis of the distribution of words in the MoCA training set to identify the most common words uttered by patients (Figure 12). We compared the top 10 words from the test data with the total distribution in the training set. The results confirm that the model could predict correct words outside the training data (Table 9).

**Figure 13:**
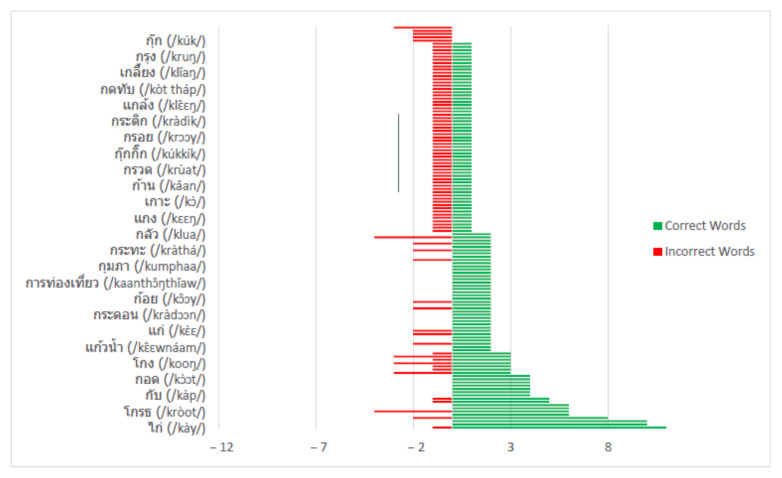
Word detection results summary.

The measurement of ASR accuracy relies on the actions of the recognizer during the decoding process. Invalid word detection is counted as a substitution error, undetected words are counted as a deletion error, and extra words generated by the decoder cause an insertion error. The detection results are reported in Table 10. The total number of incorrect words was a summation of the substitution, insertion, and deletion errors. Figure 13 highlights a summary of the total count of correct words versus incorrect words detected in our model.

### 4.4. Language Fluency Assessment

**Figure 14:**
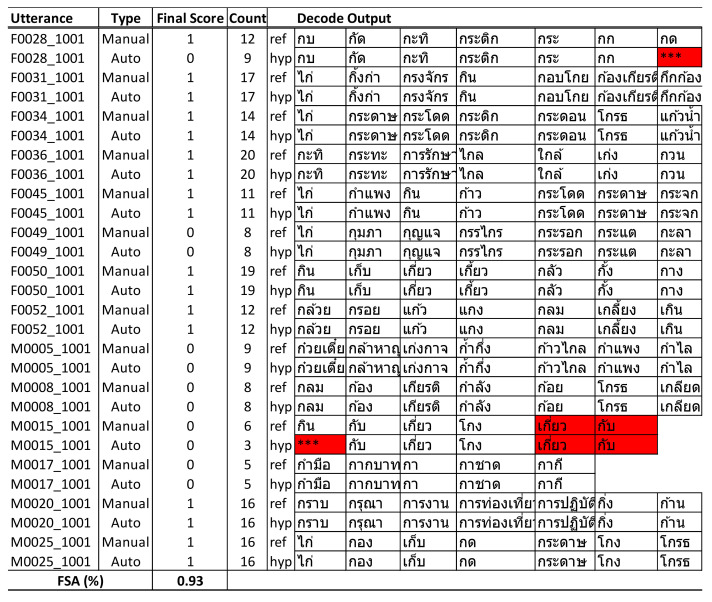

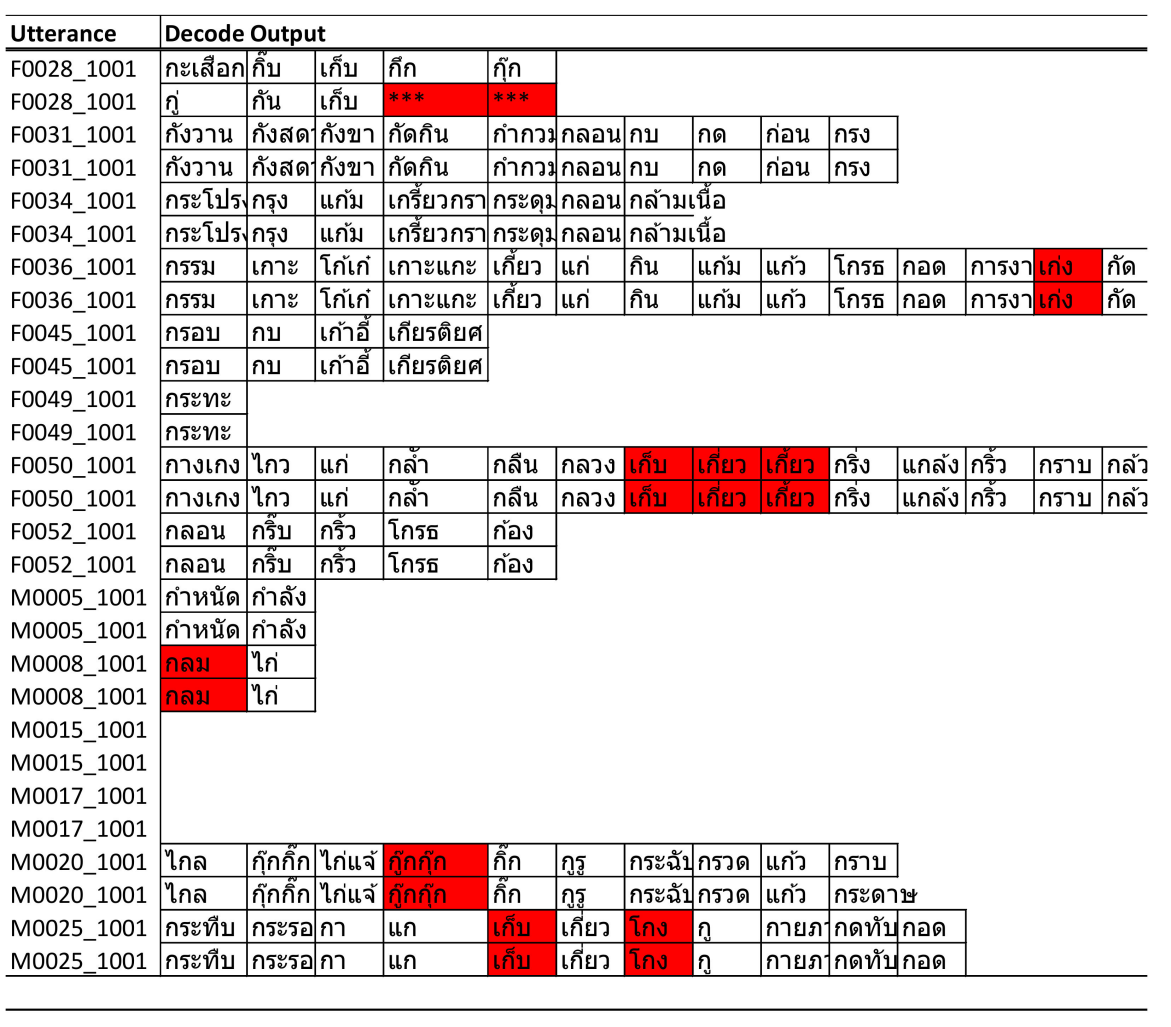
Comparison of language fluency scores between the manual and automated systems.

To evaluate the usability of an automated scoring system for the MoCA assessment tool, we examined the results of automated scoring compared with manual scoring by health professionals after the assessment. The final scoring calculated by the system shows an accuracy of 93 (Figure 14).

## 5. Discussion

### 5.1. Automatic Speech Recognition

The results of the GMM-HMM baseline analysis support our hypothesis that incorporating the LOTUS corpus could help to improve the accuracy of our model. However, an improvement in accuracy over the combined dataset produced an observation about the imbalance between the LOTUS and digital MoCA data. To enhance the reliability and generalizability of our model, an up-sampling of digital MoCA data was performed through augmentation by deploying the noise reduction and pitch shift techniques (Table 5). From the experiments, we observed that the implementation of the pitch shift algorithm on the digital MoCA data helped to reduce the deviation of cross-validation results. We used high-frequency steps to create pitch variety and simulated artificial speech as uttered by different participants in our dataset, while applying noise reduction to populate a cleaner version of speech that could improve the balance of our total dataset. This was performed to enable our model to learn and work in both noisy and quiet room environments, which helps to improve accuracy in general.

We further analyzed the results and found that the main reason for the poor results from the GMM-HMM model was a phone alignment issue that occurred during training. We used the phone alignment results from the GMM-HMM training with the LOTUS + MoCA dataset for the LF-MMI model, and the results confirm that we could not obtain a high level of accuracy because of the bad quality of input. Decoded output from the EE-LF-MMI obtained a WER of 30.77%, which was relatively high; it showed significant improvement over the other two models.

Considering that one of our main research constraints is the small amount of training data—as we are still in the developmental phase and only possess 1–2 h of speech data from MCI patients for the experiment—the initial focus was to select a method that did not require a large training dataset, coupled with the flexibility to adapt to domain-specific tasks. Therefore, based on the number of studies, GMM-HMM and DNN-HMM offer greater flexibility compared to the end-to-end approach. Furthermore, the LF-MMI achieved an optimal result over several speech recognition tasks on a relatively small corpus [29]. Therefore, we chose to apply the LF-MMI model in this study.

The end-to-end LF-MMI can obtain a comparable performance with regular LF-MMI but with a simplified training pipeline, and it works well with a small dataset. In our study, the LF-MMI approach obtained better accuracy than GMM-HMM, but the model architecture required alignment data from the previous training owing to very poor results from GMM-HMM. Thus, phone alignment information significantly affected the quality and accuracy of the TDNN layers. Meanwhile, the EE-LF-MMI approach demonstrated a high potential for future ASR development where domain-specific data are scarce with minimum effort needed for feature engineering.

### 5.2. Data Augmentation

Owing to the limited corpus of sample data from digital MoCA in our study, data augmentation emerged as the main factor for the development of our ASR model. A combination of techniques were used to populate data with a greater variety of pitch; however, the key factor involves the incorporation of the LOTUS corpus into our dataset as it contains a PD set of data for Thai phonemes that enables our acoustic model to learn the majority of the basic phone units and predict unseen words outside of the training data. The results confirm that our model can predict words outside of the training data, as shown in Table 11, which helps reduce the need for collecting a large amount of speech sample data from patients, to develop an acoustic model for a speech recognition task. To the best of our knowledge, this study was the first attempt to utilize the LOTUS corpus for supporting data augmentation and the provision of basic phone units with a limited sample of Thai speech data within the medical domain. We believe our method can contribute to the future development of Thai ASR tasks where domain-specific data are scarce.

### 5.3. Word Detection Error Analysis

We observed a slightly better accuracy rate for male speakers (70.31% correct words) compared to female speakers (69.91% correct words). We listened to the original audio recordings and found that the voice quality of male speakers seemed to be better. However, because of the small sample size, we could not draw any meaningful conclusions about the impact of model accuracy from the variation of voice quality observed between genders.

**Figure 15:**
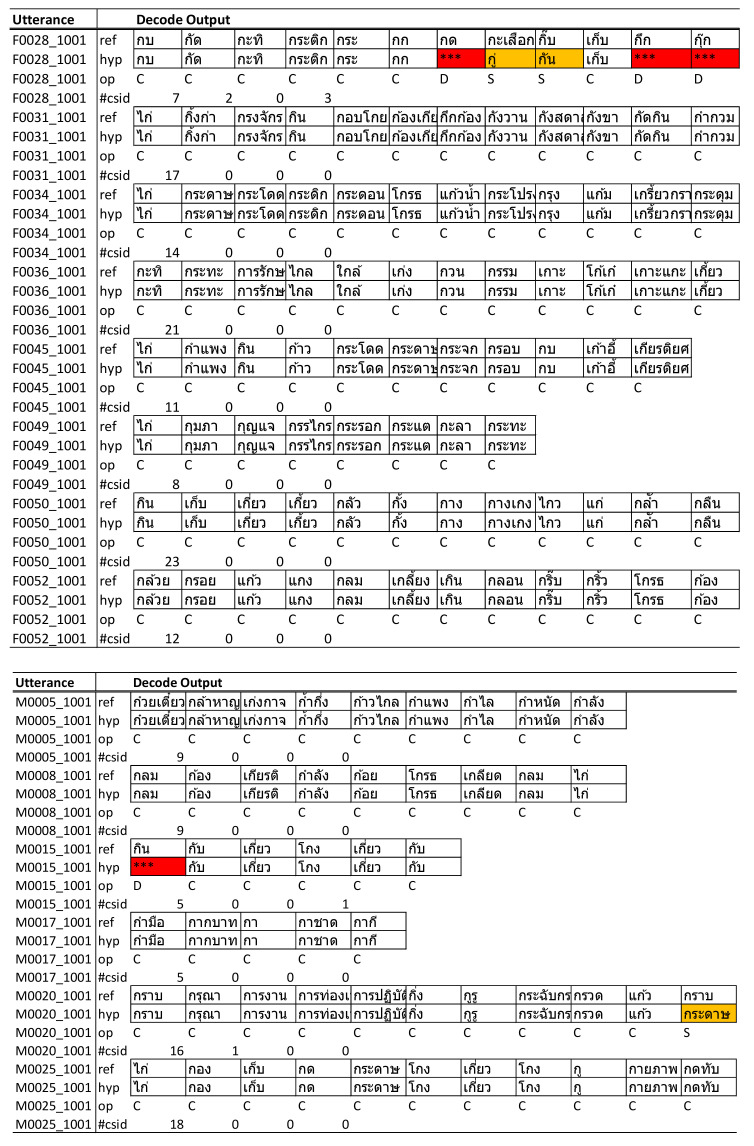
Detailed results of mismatches per utterance.

As the audio samples contained both long utterances and single words, to attain a better understanding of the sequence of errors for long utterances, we filtered out the results from single words and analyzed only long utterances. By doing so, we found that major mismatches were caused by deletion and substitution errors, as shown in Figure 15.

This result suggest that our ASR model could detect words correctly from the majority of the long utterances because of the support of the language model, while the detection of single words indicates a greater error (Table 10).

#### 5.3.1. Substitution Error

The data suggested that several of the detected words have a strikingly similar pronunciation to the test utterance. Therefore, we performed a detailed analysis of the most frequent incorrect words detected by our ASR model and found that the majority of the errors, which came from substitution errors, were caused by words of similar tone and pronunciation (Table 11 and Table 12).

**Figure 16:**
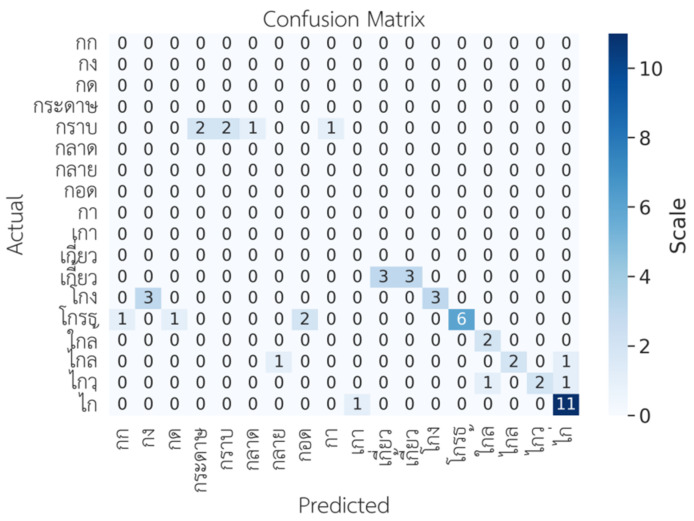
Confusion matrix for words with a similar tone.

Further analysis showed that the model could not accurately predict words with similar tones; for example, ไกล/klay/vs. ใกล้/klây/, ไกล/klay/vs. ไก่/kày/, เกี้ยว/kîaw/vs. เกี่ยว/kìaw, as shown in Figure 16.

#### 5.3.2. Deletion Error

Deletion errors occur randomly with both single words and long utterances (Table 13). We listened to the audio recordings of those words that the ASR recognizer was unable to detect and found that the majority of them contained a high level of noise, while several had issues with unclear pronunciation.

#### 5.3.3. Insertion Error

**Figure 17:**
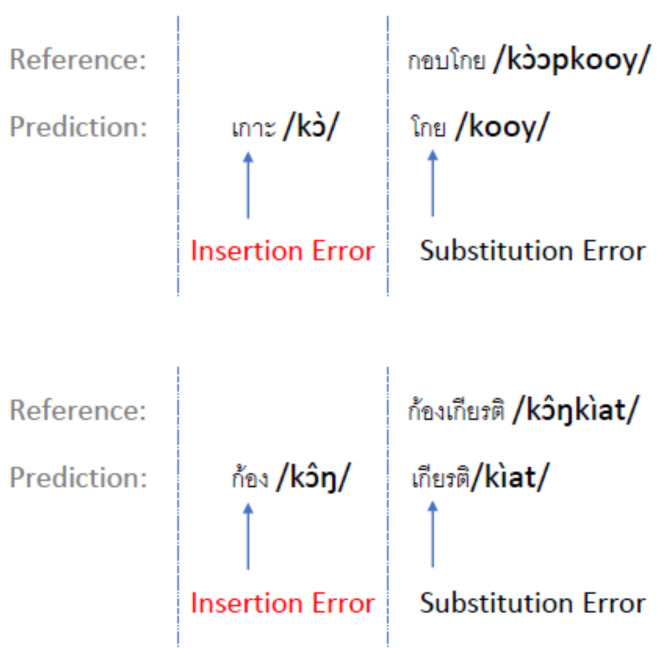
Insertion error with multiple-syllable words.

The results indicate that insertion errors occur with multiple-syllable words, where the ASR recognizer detects those syllables as separate words (Figure 17). As a consequence of the insertion error, the remaining syllables were detected as another word and caused an additional substitution error in those utterances.

### 5.4. Language Fluency Assessment

We compared the results of automated scoring with manual scoring and found that for utterances with substitution errors, the ASR recognizer attempted to predict the closest words where the initial phone started with “ก” (/k/) that were still within the dictionary. For example, the word “กราบ” (/kràap/) was incorrectly detected as “กระดาษ” (/kràdàat/); however, because of “กระดาษ”—also a valid Thai word starting with “ก” (which fully met the assessment criteria)—the scoring algorithm would count this word as eligible and result in no big deviation from the total count of words, although several of them were detected incorrectly but with no impact to the overall score for that utterance. Utterances with deletion errors would cause some words to be skipped and had a direct impact on the total word count and the overall score. However, because our ASR model had a low percentage of deletion errors in the test utterances, it had less of a negative impact on the final score.

The final score calculated by the system indicated an accuracy of 93% compared with manual scoring, as reported in Table 12. This indicates a high potential for further development and use in clinical practice to automate the scoring process of language fluency assessment.

### 5.5. Limitations and Future Directions

In this study, we developed and evaluated a new approach to integrate ASR techniques into a MoCA assessment tool for conducting a language fluency test with Thai language support. Most previous studies focus on utilizing ASR to assess verbal semantic fluency tasks in English, for which ample speech data are publicly available. This novel method is proposed to utilize ASR for assisting a phonemic fluency task in Thai, which is the first attempt of its kind. Several challenges still need to be addressed, starting from the data collection process, which directly affected the quality of voice recordings and had major impacts on the overall accuracy of ASR. Acoustic model training with low resources—that is, no domain-specific data available—was one of the biggest challenges in the speech recognition task.

The main limitation of this study is the small size of the dataset. We are in the early developmental phase of the new digital MoCA system; therefore, the Thai speech data of MCI patients are unavailable because it is the first attempt to collect data from the MoCA assessment in digital format for the Thai language. Additionally, limited numbers of iPad devices were used for the trial run; therefore, we could obtain only 1 h 40 min of data that could be used for our experiment. Consequently, it was difficult to utilize this small amount of data for the development of good ASR in general terms. Therefore, we believe that an increase in sample data will improve the overall accuracy and generalizability of our proposed ASR model. Once the digital MoCA system reaches its production stage and can be deployed for clinical use in broader communities, we will be able to collect more data for future work enhancement.

Additionally, the voice quality and control of the recording condition present challenges to our data processing steps. The data collection is performed in an open environment without the control of background noise and has a direct impact on the quality of speech recording. The majority of the utterances recorded from digital MoCA require manual data cleansing to remove the part of the conversation between health professionals and patients to ensure it contains only speech uttered by patients before its use for the training of the model. Furthermore, the current design of the digital MoCA system uses the built-in iPad microphone for voice recording; therefore, it is hard to control the voice quality and noise level. The integration of a dedicated microphone to differentiate the voices of patients from healthcare professionals facilitates the improvement of the quality of the voice recordings and reduces the need to perform the data cleansing step.

Furthermore, another limitation involves tone detection. Thai is a tonal language, and although our study implements the tone mark in the lexicon model, we observed that our ASR, in some cases, cannot accurately predict words with a similar tone. Therefore, an insufficient pitch difference can result in lower accuracy of word detection with incorrect tone, which can alter word meanings and lower the accuracy of the ASR. However, if we consider the overall language fluency score, it will not negatively impact the assessment because those detected words are approved according to the stated criteria even if they produce a different meaning. Consequently, the implementation of an algorithm for tone detection should be considered to improve the accuracy of the ASR model in the near future.

## 6. Conclusions

MoCA is a widely used tool of assessment for MCI detection, but data analysis options for the current paper-and-pencil-based version are limited and require great manual efforts in data collection. Therefore, we sought to develop digital MoCA in Thailand. This study is a sub-project of the larger digital MoCA project; the study aimed to create technology to support automatic data collection and analysis in order to enable physicians and other health professionals in Thailand to conduct reliable cognitive assessments for a broader range of patients.

This study demonstrated the possibilities of utilizing ASR techniques for word detection to assist the MoCA language fluency test scoring system for Thai. The proposed method yields acceptable accuracy under a number of constraints where domain-specific data are not publicly available. However, more data need to be collected for digital MoCA, which is still in the early phase of development. Additionally, several other challenges emerged in this study, mainly from data quality issues.

We see great potential for further improving the accuracy of the system, such as by enhancing the acoustic model with more data from patients, increasing the accuracy of the Thai tone detection with a better algorithm, and integrating a dedicated microphone system into the iPad. These points will assist in differentiating the voices of patients from health professionals, which will help to improve the quality of the voice recording. In addition to benefits within the medical domain, the techniques developed from this study, such as isolated word recognition of Thai words beginning with “ก,” can be used as a baseline for future expansion of complex ASR system integration in various speech recognition domains.

## Figures and Tables

**Table 1:** Thai consonant sounds.

Thai Consonants	Initial Consonant Sound	Final Consonant Sound	Thai Consonants	Initial Consonant Sound	Final Consonant Sound
ก	g	k	ป	bp	p
ข, ฃ, ค, ฅ, ฆ,	k	k	ผ, พ, ภ	p	p
ง	ng	ng	ฝ, ฟ	f	p
จ	j	t	ม	m	m
ฉ, ช, ฌ	ch	t	ย	y	y
ญ	y	n	ร	r	n
ด, ฎ	d	t	ล, ฬ	l	n
ถ, ฐ, ท, ธ, ฑ, ฒ	t	t	ว	w	w
ต, ฏ	Dt	t	ซ, ศ, ษ, ส, ทร	s	t
น, ณ	N	n	ห, ฮ	h	-
บ	B	p			

**Table 2:** Five meanings of khaa with different tones [12].

	Thai Script	Phonemic Transcription	English Transcription	Tone
1	คา	kh¯aa	a kind of grass	M
2	ข่า	kh`aa	galingale	L
3	ฆ่า	khˆaa	to kill	F
4	ค้า	kh´aa	to trade	H
5	ขา	khˇaa	a leg	R

**Table 3:** Details of the training dataset.

Corpus	Number of Utterances	Duration (h)
LOTUS-PD	3040	5:24
Digital MoCA	780	1:40
Augmented Digital MoCA	3900	8:24
Augmented LOTUS + MoCA	21,628	42:10

**Table 4:** Baseline ASR results.

Group	Train Dataset	Validation Dataset	K-Folds	% WER	
				AVG	SD
Baseline	MOCA	MOCA − Dev	5	86.95	23.27
	LOTUS	LOTUS	5	2.30	0.47
Augmentation	MOCA + LOTUS	MOCA + LOTUS	5	7.91	1.46
	MOCA+Pitch Shift	MOCA + LOTUS	5	87.11	5.21
	MOCA+Pitch Shift+ Noise Reduce	MOCA + LOTUS	5	80.80	10.89
	MOCA + LOTUS + AUG (Pitch Shift + Noise Reduce)	MOCA + LOTUS	5	21.20	2.90

**Table 5:** Evaluation of ASR results.

Train Dataset	Test Dataset	Features	Model	%WER
MOCA + LOTUS + AUG	MOCA	MFCC + Pitch	GMM-HMM	82.97
MOCA + LOTUS + AUG	MOCA	MFCC + iVector	TDNN-HMM (LF-MMI)	62.64
MOCA + LOTUS + AUG	MOCA	MFCC	TDNN-HMM (EE LF-MMI)	**31.87**

**Table 6:** Hyperparameter tuning for the EE-LF-MMI model.

Model	TDNN Layers	Epochs	Beam Search	Acoustic Weight	%WER
Baseline	13	10	15	1	31.87
EE-LF-MMI-1	13	5	15	1	31.87
EE-LF-MMI-2	15	5	15	1	32.42
EE-LF-MMI-3	9	5	15	1	34.89
EE-LF-MMI-4	13	5	15	0.8	**30.77**
EE-LF-MMI-5	13	5	10	0.8	32.69

**Table 7:** Comparison of accuracy impact by data augmentation over combined dataset.

Train Dataset	Test Dataset	%WER
MOCA	MOCA	95.05
MOCA+Pitch Shift	MOCA	89.94
MOCA+Pitch Shift+Noise Reduce	MOCA	86.54
MOCA + LOTUS + AUG (Pitch Shift+Noise Reduce)	MOCA	82.97

**Table 8:** Statistics of the recognizer results by speaker.

Speaker	ID	#Word	Corr	Sub	Ins	Del	Err
F0028	raw	24	8	8	0	8	16
F0028	sys	24	33.33	33.33	0	33.33	66.67
F0031	raw	34	25	7	2	2	11
F0031	sys	34	73.53	20.59	5.88	5.88	32.35
F0034	raw	28	19	4	0	5	9
F0034	sys	28	67.86	14.29	0	17.86	32.14
F0036	raw	42	34	8	1	0	9
F0036	sys	42	80.95	19.05	2.38	0	21.43
F0045	raw	22	17	3	0	2	5
F0045	sys	22	77.27	13.64	0	9.09	22.73
F0049	raw	16	12	4	0	0	4
F0049	sys	16	75	25	0	0	25
F0050	raw	46	34	12	0	0	12
F0050	sys	46	73.91	26.09	0	0	26.09
F0052	raw	24	16	8	0	0	8
F0052	sys	24	66.67	33.33	0	0	33.33
M0005	raw	18	12	6	0	0	6
M0005	sys	18	66.67	33.33	0	0	33.33
M0008	raw	18	14	4	0	0	4
M0008	sys	18	77.78	22.22	0	0	22.22
M0015	raw	12	9	2	0	1	3
M0015	sys	12	75	16.67	0	8.33	25
M0017	raw	10	7	3	0	0	3
M0017	sys	10	70	30	0	0	30
M0020	raw	34	22	11	0	1	12
M0020	sys	34	64.71	32.35	0	2.94	35.29
M0025	raw	36	26	8	0	2	10
M0025	sys	36	72.22	22.22	0	5.56	27.78
SUM	raw	364	255	88	3	21	112
SUM	sys	364	70.05	24.18	0.82	5.77	30.77

**Table 9:** Top 10 words from test utterances.

Word	IPA	Count	Word Count in Training	Correctly Detected
ไก่	kày	12	0	11
เก็บ	kèp	10	0	10
เกี่ยว	kìaw	10	0	10
โกรธ	kròot	10	0	6
กิน	kin	10	245	8
เกี้ยว	kîaw	6	0	3
แก้ว	kɛ̂ɛw	6	0	6
โกง	kooŋ	6	0	3
กด	kòt	6	50	3
กบ	kòp	6	120	5

**Table 10:** Detection result of test utterances.

	Single Words		Long Utterances	
Detection Result	Count	Percent	Count	Percent
Correct	80	44%	175	96%
Substitution	82	45%	3	2%
Deletion	17	9%	4	2%
Insertion	3	2%	0	0%

**Table 11:** Example of substitution error detected by ASR.

Error	Ref	IPA	Detect	IPA	Ref	IPA	Detect	IPA
Substitution	โกง	kooŋ	กง	koŋ	กอง	kɔɔŋ	ก้อง	kɔ̂ŋ
Substitution	เกี้ยว	kîaw	เกี่ยว	kìaw	กาง	kaaŋ	ก้าง	kâaŋ
Substitution	กราบ	kràap	กระดาษ	kràdàat	กิน	kin	เก่ง	kèŋ
Substitution	โกรธ	kròot	กอด	kɔ̀ɔt	แกง	kɛɛŋ	ก้อง	kɔ̂ŋ
Substitution	กก	kòk	กบ	kòp	แก่	kɛ̀ɛ	ก้อง	kɔ̂ŋ
Substitution	กด	kòt	กิน	kin	แก่	kɛ̀ɛ	แก้ว	kɛ̂ɛw
Substitution	กบ	kòp	กง	koŋ	ไกล	klay	ไก่	kày
Substitution	แก	kɛɛ	แก่	kɛ̀ɛ	ไกล	klay	กลาย	klaay
Substitution	กรง	kroŋ	ก้อน	kɔ̂ɔn	ไกว	kway	ไก่	kày
Substitution	กระ	krà	กลับ	klàp	ไกว	kway	ใกล้	klây

**Table 12:** Most frequent incorrect words detected by ASR.

Error	Ref	IPA	Detect	IPA	Count
Deletion	กด	kòt	***		2
Substitution	โกง	kooŋ	กง	koŋ	3
Substitution	เกี้ยว	kîaw	เกี่ยว	kìaw	3
Substitution	กราบ	kràap	กระดาษ	kràdàat	2
Substitution	โกรธ	kròot	กอด	kɔ̀ɔt	2
Substitution	กด	kòt	กิน	kin	1
Substitution	กราบ	kràap	กา	kaa	1
Substitution	กราบ	kràap	กลาด	klâat	1
Substitution	โกรธ	kròot	กก	kòk	1
Substitution	โกรธ	kròot	กด	kòt	1

**Table 13:** List of words that could not be detected by ASR.

Ref	Prediction	Count	Ref	Prediction	Count
เกรี้ยวกราด	***	1	กังขา	***	1
เกียรติยศ	***	1	กัด	***	1
แก้ม	***	1	ก้าว	***	1
กด	***	1	กิ๊บ	***	1
กระดุม	***	1	กึก	***	1
กระรอก	***	1	กึกก้อง	***	1
กรุง	***	1	กุ๊ก	***	1
กล้ามเนื้อ	***	1	กุ๊กกิ๊ก	***	1
กะเสือกกะสน	***	1			
